# Cross-Sectional Analysis of Sleep Quality and Vascular Health in Shift- and Day-Working Nurses

**DOI:** 10.3390/clockssleep7040058

**Published:** 2025-10-11

**Authors:** Gleb Saharov, Barbara Salti, Maram Bareya, Anat Keren-Politansky, Yona Nadir, Tamar Shochat

**Affiliations:** 1 Thrombosis and Hemostasis Unit, Rambam Health Care Campus, Haifa 3109601, Israel; g_sakharov@rambam.health.gov.il (G.S.); b_muller@rambam.health.gov.il (B.S.); m_bareya@rambam.health.gov.il (M.B.); anat_keren@rambam.health.gov.il (A.K.-P.); y_nadir@rambam.health.gov.il (Y.N.); 2 Department of Nursing, Faculty of Social Welfare and Health Sciences, University of Haifa, Haifa 3109601, Israel

**Keywords:** endothelial dysfunction, coagulation, hemostatic, total sleep time, sleep efficiency, social jetlag

## Abstract

Sleep disturbances and shift work are associated with increased cardiovascular risk, possibly through disruptions in endothelial and hemostatic function. While prior studies link acute sleep deprivation to vascular dysfunction, the impact of chronic sleep quality and circadian misalignment on endothelial health in healthy individuals, particularly shift workers, remains underexplored. The aim of this study was to examine the association between objectively measured sleep quality and endothelial/hemostatic function in healthy female hospital nurses, comparing shift and day workers, and considering time-of-day variation. In this repeated-measures study, 100 female nurses (51 shift, 49 day workers) aged 25–50 wore actigraphy devices for 7–14 days to assess total sleep time (TST), sleep efficiency (SEF), and wake after sleep onset (WASO). Endothelial function was measured using EndoPAT (Reactive Hyperemia Index—RHI). Hemostatic markers included plasminogen activator inhibitor-1 (PAI-1), von Willebrand factor (VWF), heparanase and heparanase procoagulant activity assessed by ELISA, and chromogenic assays in morning and evening. TST was not associated with any vascular outcomes. Poor sleep quality (low SEF, high WASO) was significantly associated with reduced RHI and elevated PAI-1 level, heparanase level, and heparanase procoagulant activity levels. Regression models revealed significant main effects of SEF and WASO on endothelial and coagulation markers, with some interactions depending on shift type and time of measurement. No significant associations were found for VWF. Impaired sleep quality, but not sleep duration, is associated with endothelial dysfunction and procoagulant activation, particularly among shift-working nurses. These findings suggest that sleep quality may play a critical role in vascular health and support the use of sleep-based interventions to reduce cardiovascular risk in shift-working populations.

## 1. Introduction

Epidemiologic studies indicate a link between sleep disturbances, including short [1] and long [2] sleep duration, sleep apnea [3], insomnia [4] as well as mistimed sleep [5], and an increased risk of cardiovascular disease (CVD). Biological pathways implicated in the association between sleep disturbance and CVD include cardiovascular autonomic control, oxidative stress, inflammatory responses, and endothelial dysfunction [6]. Notably, most of the evidence is based on acute sleep deprivation, whereas the evidence for the effects of chronic sleep deprivation in healthy individuals is lacking. Mistimed sleep has also been shown to increase cardiometabolic risk factors independently of sleep disturbance [7]. Shift work, which is associated with mistimed and inadequate sleep, has also been shown to be a risk factor for CVD [8], yet the underlying mechanisms are yet to be fully understood.

The hemostatic system has a significant impact on cardiovascular morbidity [9]. Impaired endothelial function is an early sign of vascular abnormality preceding clinically overt cardiovascular disease [10]. The intact endothelium regulates vascular tone and repair capacity, maintaining pro-inflammatory, anti-inflammatory, and coagulation homeostasis. Alteration of these homeostatic pathways results in endothelial dysfunction before structural changes in the vasculature [11].

The relationship between sleep and the balance of endothelial function has been explored in various studies [12,13]. Based on a systematic review of 24 studies, sleep deprivation, including short sleep, restricted sleep, acute total sleep deprivation, sleeping outside the 7–9 h range, and deprived sleep associated with shift work are all associated with micro- and/or macro-vascular endothelial function [13]. However, these studies are typically between-subjects designs and are based on single-assessment methods for both sleep and endothelial function. Sleep measurements that have been used include polysomnography [14], actigraphy [12], and subjective sleep assessments [15]; whereas endothelial function is typically measured as peripheral arterial tonometry [14], heart rate variability [16], or brachial artery flow-mediated dilation (FMD) [15].

Furthermore, few have examined these associations in the context of chronic shift work. A recent scoping review has suggested that the evidence of an effect of shift work on endothelial function is scarce [17]. We have previously shown, in a comparative cross-sectional study design, that shift-working female hospital nurses, likely not at risk for cardiovascular disease, demonstrated higher coagulation markers than their day-working counterparts. These associations were partially explained by sleep quality [18].

Endothelial function and hemostatic markers exhibit diurnal variation, with morning peaks in prothrombotic activity and reduced vascular reactivity that improve later in the day [19]. To capture the dynamic impact of sleep disruption on vascular health, endothelial function was assessed both in the morning and evening. Measuring in both the morning and evening enables the detection of time-specific effects of sleep quality and shift work on vascular health, enhancing physiological interpretation [6,19] and ensuring that abnormal endothelial dynamics across the day were not overlooked. Evaluating two time points allows for differentiation between transient, sleep-related effects and persistent endothelial dysfunction, providing greater physiological insight than single measurements. This approach is particularly relevant because circadian misalignment and poor sleep quality can exacerbate morning endothelial impairment and coagulation risk [6].

In order to further understand this association between endothelial and coagulation dynamics and their role in the link between sleep disturbances and cardiovascular morbidity in healthy individuals, including shift workers, it is essential to use a range of assessment tools—such as repeated measurements at different time points and a variety of endothelial and hemostatic markers.

The main objective of the present study was to assess the contribution of sleep duration measured as total sleep time (TST) and quality measured by sleep efficiency (SEF) and wake after sleep onset (WASO) on endothelial and hemostatic functions in healthy shift-working and day-working hospital nurses, at two time points, morning and evening. We hypothesized that poor sleep quality and shorter sleep duration are associated with impaired endothelial and hemostatic markers, and that these relationships are moderated by time of day (morning/evening hemostatic measurements) and work schedule (shift/day work).

## 2. Results

Sleep parameters were approximately normally distributed as assessed by skewness and kurtosis. The coagulation tests were also approximately normally distributed except for heparanase procoagulant activity (HPA). A log transformation of HPA was performed in order to make the data more symmetric. The EndoPAT logarithmic Reactive Hyperemia Index (LnRHI) was used to normalize the skewed distribution of linear RHI values.

One hundred nurses (mean age 39.6 ± 7.6) participated. Out of the total sample of 100 nurses, 9 were menopausal—6 from the day shift group and 3 from the shift work group. Sleep episodes per 24 h were recorded for an average of nine consecutive days (SD: 2.6; range 4–14). A total of 15 participants had follow-up periods shorter than 7 days (ranging from 4 to 6 days). Of these, two participants had data for four days due to a technical malfunction in the watch, while thirteen participants wore it for five–six days due to compliance issues. Per all 873 sleep episodes, TST ranged from 1.15 to 15.8 h (mean 6.43 h, SD:1.84 h), SEF ranged from 54.8% to 99.9% (mean 85.5%, SD: 6.1%), WASO ranged from 0 to 131 min (mean 33.5 min., SD:17.3 min). Over all nurses, 28% slept less on average than 6 h a night (12 day workers, 16 shift workers), 42% had SEF of less than 85% (14 day workers, 28 shift workers), and 93% had WASO greater than 20 min [60% more than 30 min WASO].

Table 1 presents the sleep parameters for all nurses by work schedule (shift/day). There was no statistically significant difference in average TST between shift and day workers (mean difference of 6 min). Average SEF was lower in shift compared to day workers (mean difference: −2.06%; se: 0.7%; *p* = 0.05, 95% CI: −3.5 to −0.6%) but this was not clinically meaningful. Similarly, there was no statistically significant difference in average WASO (mean difference 2.9 min, *p* = 0.06). After adjusting for age, there was no significant difference in SEF (F (1,97) = 1.29, *p* = 0.26).

### 2.1. Correlations Between Sleep Parameters and Endothelial Markers for Whole Sample

Significant correlations were maintained following FDR for correlations between sleep parameters and endothelial markers for the whole sample. Pearson correlations (Table 2) revealed that TST was not correlated with any endothelial marker. SEF was statistically significantly positively correlated with both morning and evening RHI and statistically significantly negatively correlated with both morning and evening PAI-1, Heparanase ELISA and HPA. WASO was negatively correlated with morning and evening RHI and positively correlated with both morning and evening PAI-1 and Heparanase ELISA and was not correlated with heparanase procoagulant activity.

**Figure 1:**
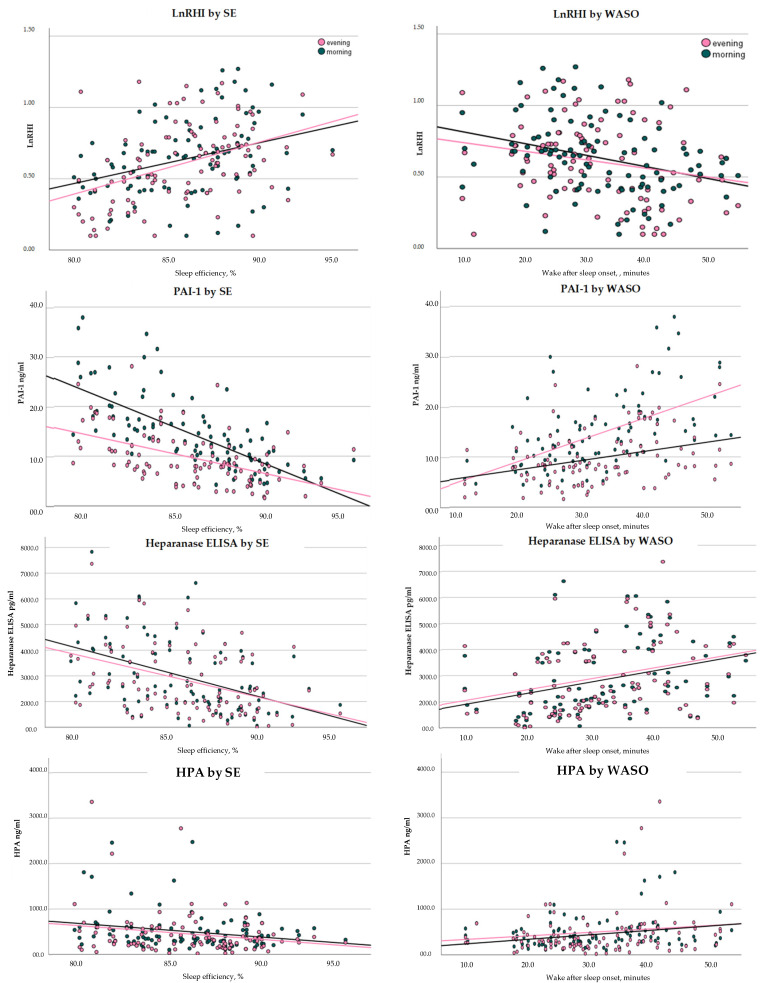
Correlations between SE, WASO, and endothelial markers. Abbreviations: WASO = Wake After Sleep Onset; SE = Sleep Efficiency; LnRHI = Natural logarithm of Reactive Hyperemia Index; PAI-1 = Plasminogen Activator Inhibitor-1; HPA = Heparanase procoagulant activity.

After correcting for age, SEF was positively correlated (see Figure 1) with both morning and evening RHI (r = morn: r = 0.290, *p* = 0.004; even: r = 0.355, *p* < 0.001) and negatively correlated with both morning and evening PAI-1 (morn: r = −0.696, *p* < 0.001; even: r = −0.529, *p* < 0.001) and heparanase (morn: r = −0.389, *p* < 0.001; even: r = −0.338, *p* < 0.001) and heparanase procoagulant activity (HPA) (morn: r = −0.391, *p* < 0.001; even: r = −0.361, *p* < 0.001). WASO was positively correlated with both morning and evening PAI-1 (morn: r = 0.575, *p* < 0.001; even: r = 0.385, *p* < 0.001), Heparanase ELISA (morn: r = 0.334, *p* < 0.001; even: r = 0.331, *p* < 0.001) and HPA (morn: r = 0.340, *p* < 0.001; even: r = 0.355, *p* < 0.001).

As TST was not correlated with any of the endothelial markers, regression models were performed only with SEF and WASO.

### 2.2. Multiple Regression Models

#### 2.2.1. RHI (Logarithmic LnRHI)

SEF: A main effect was found for SEF, in which increased SEF was significantly associated with increased average RHI. Although the time by SEF interaction was not statistically significant, it should be noted that an association between SEF and RHI was found in the evening RHI (b = 0.029, se: 0.011, *p* = 0.011, 95% CI: 0.007 to 0.051) but not morning RHI (b = 0.018, se: 0.011, *p* = 0.10, 95% CI: −0.004 to 0.041). For every 5% increase in SEF, evening RHI increased by 0.145.

No main effects were found for time of measurement or shift pattern, and no 2- or 3-way interactions were found.

WASO: A significant main effect was found for WASO so that increased WASO was associated with decreased average RHI (b= −0.005, se: 0.002, *p* = 0.010, 95% CI: −0.009 to −0.001). For every 20 min increase in WASO RHI decreased by 0.100 units.

No main effects were found for time of measurement or shift pattern, and no 2- or 3-way interactions were found.

#### 2.2.2. PAI-1

**Figure 2:**
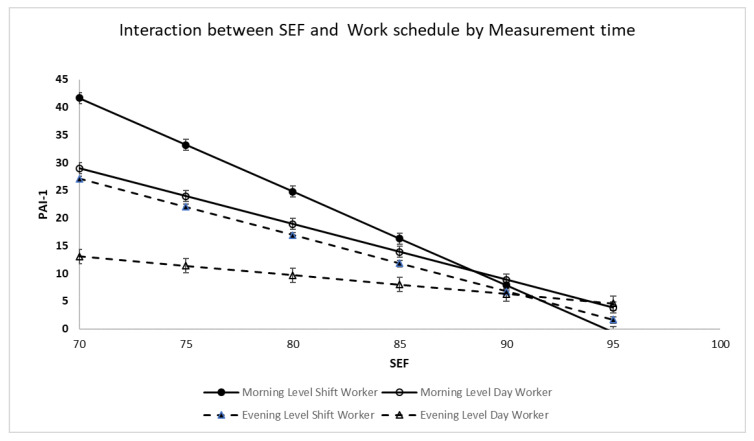
PAI-1: Interaction between SEF and Work schedule by Measurement time. The figure was derived from the multiple regression model. The bars represent the standard error from the model.

SEF: Main effects were found for time of measurement, shift pattern, and SEF; there was a two-way interaction between time of measurement and SEF and shift by SEF, but no three-way interaction between time of measurement, shift, and SEF. Post hoc analysis (see Figure 2) revealed that for day workers, both morning PAI-1 (b = −1.004, se: 0.161, *p* < 0.001, 95% CI: −1.328 to −0.681) and evening PAI-1 (b = −0.344, se: 0.148, *p* = 0.024, 95%, CI: −0.642 to −0.047) declined with increasing SEF. Among day workers, for every 5 percent increase in SEF morning PAI-1 decreased by 5.02 units whereas evening PAI-1 decreased by 1.72 units. Morning PAI-1 slope was statistically significantly different as compared to evening PAI-1 slope (t = 3.02, *p* = 0.003). Increasing SEF had a greater impact on PAI-1 decrease in the morning as compared to the evening.

Similarly, for shift workers, both morning PAI-1 (b = −1.686, se: 0.240, *p* < 0.001, 95% CI: −2.169 to −1.202) and evening PAI-1 (b = −1.054, se: 0.188, *p* < 0.001, 95% CI: −1.432 to −0.675) declined with increasing SEF. Among shift workers, for every 5 percent increase in SEF morning PAI-1 decreased 8.43 units whereas evening PAI-1 decreased by 5.27 units. Morning PAI-1 decline was statistically significantly different as compared to evening PAI-1 (t = 2.07, *p* = 0.039).

WASO: Main effects were found for time of measurement, shift pattern, and WASO. In addition, there were two-way interactions between time of measurement and WASO and shift pattern and WASO. There were no three-way interactions. For morning PAI-1, a main effect was found for WASO (b = 0.216, se: 0.099, *p* = 0.033, 95% CI: 0.02 to 0.41) and there was an interaction between shift pattern and WASO (night shift b = 0.309, se: 0.124, *p* = 0.014, 95% CI: 0.064 to 0.56). For day workers a 20 min increase in WASO resulted in a 4.32-unit increase in morning PAI-1 whereas in night shift workers a 20 min increase in WASO resulted in a 2.32-unit increase in morning PAI-1.

For evening PAI-1, an interaction was found between shift pattern and WASO (Night shift: b = 0.265, se: 0.098, *p* = 0.008, 95% CI: 0.07) such that a 20 min increase in WASO resulted in a 0.32-unit increase in evening PAI-1. There was no association between WASO and evening PAI-1 for day workers.

### 2.3. Heparanase ELISA

There were no differences between morning and evening Heparanase ELISA measurements.

SEF: A main effect was found for SEF. There was no SEF by shift interaction. Increased SEF was significantly associated with a decrease in Heparanase ELISA (b = −134.70, se = 34.74, *p* < 0.001, 95% CI: −203.65 to −65.74). For every 5% increase in SEF Heparanse Elisa decreased by 673.5 (pg/mL).

WASO: A main effect was found for WASO. There was no WASO by shift interaction. Increased WASO was significantly associated with an increase Heparanase ELISA (b = 44.08, se = 12.41, *p* < 0.001, 95% CI: 19.44 to 68.71). A 20 min increase in WASO was associated with an 881.6 (pg/mL) unit increase in Heparanase ELISA.

### 2.4. Heparanase Procoagulant Activity (HPA)

There were no differences between morning and evening (log) Heparanase Procoagulant Activity measurements.

SEF: A main effect was found for SEF. There was no interaction between shift and SEF. A 5% increase in SEF resulted in a −0.10 decrease in the log of HPA (b = −0.020, se: −0.03, to −0.01, *p* = 0.005).

WASO: A main effect was found for WASO; there was no 2-way interaction between shift and WASO. Increased WASO was significantly associated with increased HPA (b = 0.007, se: 0.002, *p* < 0.001, 95% CI: 0.003 to 0.010). A 20 min increase in WASO was associated with a 0.14 increase in the log of HPA.

VWF: There was no main effect of time, shift type nor was there a main effect of either of the sleep variables.

## 3. Discussion

The present research explores for the first time the associations between sleep parameters and vascular function measured in the morning and evening considering possible diurnal variation in endothelial and coagulation markers and using different methods of evaluation of endothelial function. We hypothesized that an association would be observed between sleep measures and endothelial coagulation markers, reflecting a potential link between sleep quality and vascular health. Our findings support this hypothesis and provide important insights into the relationship between sleep and endothelial function. In line with study hypotheses, we found associations between sleep measures and endothelial and coagulation markers, and these associations interacted with work schedules (shift/day), and time-of-day of the measurements (morning/evening).

First, we found that poor sleep quality, specifically SEF and WASO, were associated with impaired endothelial and coagulation markers such as reactive hyperemia index (RHI), plasmin activator inhibitor-1 (PAI-1), and heparinase markers that reflect both functional and structural changes in vascular health. Findings support a cross-sectional study that reported that lower actigraphy based sleep efficiency (but not sleep duration or subjective sleep quality) was associated with impaired endothelial function measured by brachial FMD [20]. The current study extends previous findings by demonstrating that impaired sleep quality (low SEF, high WASO) correlates with endothelial dysfunction and elevated PAI-1 and heparanase, but not vWF, in healthy nurses. This aligns with prior evidence linking poor sleep with increased PAI-1 and procoagulant activity [21,22] and supports the role of disrupted sleep in promoting hypercoagulability [23]. However, unlike the mixed biomarker findings reported in mid-life women [24], our results emphasize sleep quality over duration, highlighting the potential value of targeting sleep consolidation in vascular risk mitigation.

Endothelial function as measured by the Reactive Hyperemia Index (RHI, EndoPAT) showed a reduction in vascular responsiveness in individuals with impaired sleep, and such a tendency was observed in both the morning and evening measurements. Higher SEF was significantly linked to increased evening RHI, suggesting better endothelial function later in the day among individuals with more consolidated sleep. Although the time by SEF interaction was not statistically significant overall, the evening-specific association (b = 0.029, *p* = 0.011) indicates that improvements in sleep efficiency may have a more pronounced benefit on vascular reactivity during the evening compared to the morning. This aligns with emerging evidence showing that sleep quality, particularly efficiency, plays a crucial role in cardiovascular health through mechanisms involving endothelial function [25], and even mild sleep restriction may increase endothelial oxidative stress in female persons [26]. Conversely, increased WASO was associated with decreased average RHI (b = −0.005, *p* = 0.010), further reinforcing that fragmented sleep may impair endothelial responsiveness, potentially elevating cardiovascular risk.

PAI-1, as a fibrinolysis suppressor and prothrombotic enzyme, was negatively correlated with sleep efficiency. The present findings, as in our previous study [18,27], underscore an association between sleep quality parameters, SEF and WASO, and levels of plasminogen activator inhibitor-1 (PAI-1), with both circadian timing and shift schedule playing significant moderating roles. Both day- and shift-working nurses showed statistically significant declines in PAI-1 levels with increasing SEF, with a stronger effect observed in the morning samples. Notably, a 5% increase in SEF was associated with a reduction of 5.02 units in morning PAI-1 among day workers and 8.43 units among shift workers, indicating greater vulnerability of shift workers to sleep-related hemostatic dysregulation. These findings are consistent with prior studies reporting elevated PAI-1 levels among shift-working nurses compared to daytime workers, suggesting subclinical coagulation activation likely due to circadian misalignment and chronic sleep disruption [18,27,28]. Similarly, increased WASO was positively associated with morning PAI-1, particularly in night-shift nurses, further supporting a link between fragmented sleep and prothrombotic changes. Elevated PAI-1 levels in the morning—especially among shift workers—highlight the role of disrupted sleep and circadian misalignment in creating a prothrombotic state [28]. These hemostatic responses of PAI-1 to poor sleep may represent early biomarkers of increased cardiovascular risk in this occupational group [19], warranting further investigation into interventions that promote sleep continuity and circadian alignment in shift-working nurses. These PAI-1 findings were consistent with our previous report [18] when we described the association between coagulation markers and sleep quality.

Heparanase, a marker of endothelial glycocalyx degradation, was significantly higher in shift workers during both time points, reinforcing our hypothesis that sleep insufficiency may contribute to endothelial damage. In this study, both Heparanase ELISA and Heparanase Procoagulant Activity (HPA) were significantly associated with sleep parameters SEF and WASO. Increased SEF was significantly linked to lower Heparanase ELISA levels and reduced HPA, suggesting that better sleep continuity may suppress heparanase expression and its procoagulant effects. Conversely, higher WASO, indicative of more fragmented sleep, was associated with elevated levels of both Heparanase ELISA and HPA, reinforcing the role of disrupted sleep in enhancing procoagulant activity. These findings are consistent with previous research indicating elevated heparanase levels in shift-working nurses compared to daytime workers, potentially contributing to increased cardiovascular risk in this population [18]. Experimental studies have also shown that sleep fragmentation increases sympathetic nervous system activity [29] and cortisol levels [30,31], promoting metabolic [32] and hemostatic dysregulation [33]. These physiological stress responses could upregulate heparanase, known to enhance tissue factor activity and initiate coagulation cascades. Together, these findings underscore the potential mechanistic link between impaired sleep and heightened coagulation risk via heparanase pathways, emphasizing the need for targeted sleep interventions among health care shift workers to mitigate cardiovascular risk.

These results align with previous research [18,27] suggesting that poor sleep may contribute to endothelial and coagulation dysregulation, potentially mediated through increased inflammatory [34] and coagulation responses [23]. Sleep duration (TST) was not in any association with endothelial and coagulation markers at any time of day.

In line with previous findings, elevated levels of von Willebrand factor (VWF)—a large multimeric glycoprotein secreted by endothelial cells involved in platelet adhesion—may reflect endothelial activation [35] and an increased prothrombotic state, highlighting a potential mechanism contributing to elevated cardiovascular risk [36]. Surprisingly, no significant associations were found for sleep parameters and VWF in our present study. These discrepancies may be due to small sample size and due to the relatively young age of participants (39.6 ± 7.6). However general population studies have described a weak association between plasma VWF and the risk of CVD [37], while on the other hand it is considered a significant risk marker for CVD in older adults [38].

The findings of multiple regression underscore the significance of sleep efficiency in influencing vascular and procoagulant biomarkers among nurses. Higher SEF was associated with improved endothelial function. This aligns with recent research suggesting that better sleep continuity enhances cardiovascular recovery and reduces pro-thrombotic states [39]. Conversely, increased sleep fragmentation (WASO) was consistently linked to adverse outcomes, including decreased RHI and elevated PAI-1, Heparanase, and HPA levels, which are all markers of impaired vascular health and heightened thrombotic risk. These results are supported by prior evidence indicating that fragmented sleep is associated with systemic inflammation [40] and endothelial dysfunction [41,42]. The differential effects observed between morning and evening measurements suggest potential diurnal variability in biomarker responses to sleep parameters, emphasizing the importance of temporal context in interpreting physiological data.

By focusing on healthy participants, the study establishes a baseline understanding of physiological norms, enabling earlier detection of endothelial dysfunction related to sleep disturbances. This could lead to innovative screening tools or lifestyle interventions aimed at improving cardiovascular health.

However, it is important to acknowledge the limitations of our study. These include the small sample size, with a specific focus only on healthy female nurses. In addition, 93% of the nurses (shift and day workers) had WASO over 20 min, indicating that most of the sample had poor sleep quality. This calls into question how representative the sample was, or alternatively, whether the 20 min benchmark for WASO should be used in this setting. Moreover, sleep efficiency was measured at the time of or after blood collection. To establish causality, sleep measurements should have been performed prior to blood collection. However, sleep measurements were performed in nurses’ ecological settings, and we considered them to represent their typical sleep.

Another limitation of our study is that our cohort of 100 healthy women included 9 postmenopausal participants; however, we did not perform stratified analysis by menopausal status due to the small subgroup size. While their inclusion may have contributed to variability in endothelial and coagulation markers, the present results should be interpreted primarily in the context of overall sleep-related effects. In addition, we did not account for the potential influence of menstrual cycle phase among premenopausal participants, which is known to affect both sleep characteristics and vascular function, possibly introducing further variability into our findings. Future studies with larger samples of postmenopausal women and with careful consideration of menstrual cycle phase are warranted to disentangle the overlapping contributions of sleep disruption, estrogen deficiency, and hormonal fluctuations to endothelial dysfunction and prothrombotic changes.

## 4. Materials and Methods

Study design: The study was a prospective repeated-measures design, comparing hemostatic and endothelial markers, measured in the morning and evening, and sleep duration and quality, measured over 7–14 days, in female hospital nurses working shift- or day-work schedules.

Participants: The study was approved by the Institutional Research Ethics Committee of Rambam Health Care Campus, No. 0303-18-RMB. Inclusion criteria consisted of female nurses age 25–50 years, body mass index (BMI) ≤ 30, and working at least 75% of full-time job (32 h per week) on either regular day work or rotating day–evening–night shifts. Exclusion criteria were the presence of diabetes, hypertension, pregnancy or postpartum period, and use of any chronic medications (excluding hormonal replacement treatments). Based on these criteria, nurses were identified from the Rambam Health Care Campus staff database and convenience sampling was conducted by sending out information via internal hospital communication platforms, WhatsApp, and personal correspondence. Volunteers completed a screening questionnaire including age, weight, height, chronic illness, pregnancy status, and total experience in nursing. Quota sampling was completed after recruitment of 105 nurses (53 shift workers, 52 day workers). Five nurses were subsequently excluded from analysis due to COVID-19 infection. All participants signed written informed consent.

### 4.1. Data Collection Tools

Sociodemographic, employment, and health characteristics: The sociodemographic/health questionnaire prepared by the researchers included age, type of hospital shift, total work experience, full/part time job, and BMI based on current weight and height.

Sleep parameters: For objective assessment of sleep, an actiwatch (Actiwatch Spectrum; Respironics Inc., Murrysville, PA, USA) was used concurrently with daily sleep logs to determine time spent in bed.

The sleep log included sleep onset, duration, and wake time. Activity data was downloaded to the Phillips Actiware-6 software and analyzed for parameters including total sleep time (TST), sleep efficiency (SE), and wake after sleep onset (WASO).

### 4.2. Endothelial and Coagulation Evaluation

Participant preparation: Participants were instructed to fast for at least 4 h before the EndoPAT test, avoid caffeine, alcohol, and smoking for at least 8 h, and refrain from strenuous physical activity for 24 h. All tests were conducted in the same room, which was darkened, and any noises such as phones, intercoms, or monitor alarms were muted. Participants rested in a supine position for 15 min before and during testing to ensure stable baseline vascular conditions.

Endothelial function assessment: To assess endothelial status, we performed noninvasive endothelial function tests with the EndoPAT system (Itamar Medical Ltd. Caesarea, Israel), a US Food and Drug Administration (FDA)-cleared non-invasive test to evaluate the level of endothelial function. EndoPAT assesses digital-flow-mediated dilation during reactive hyperemia using measurements from both arms—occluded side that is applied by near-diastolic external pressure and control side [43,44]. EndoPAT provides an endothelial function Reactive Hyperemia Index (RHI) that is the post-to-pre-occlusion signal ratio in the occluded side, normalized to the control side. Reactive hyperemia is a well-established technique for noninvasive assessment of peripheral microvascular function and a predictor of all-cause cardiovascular morbidity and mortality [45]. Low index indicates an endothelial dysfunction.

Blood coagulation parameters: Ten milliliters of blood were drawn to 3.2% citrated tubes at 6:00–9:00 and 18:00–21:00, immediately after the EndoPAT assessment was performed. Plasma was obtained by centrifugation (1500 g for 15 min at room temperature). All coagulation assays were performed on the thawed frozen plasma samples. Plasma samples were frozen after a second centrifugation at 2000× *g* for 15 min in aliquots, at −70 ± 5 °C. Prior to testing, plasma aliquots were thawed in a 37 ± 0.5 °C water bath for 15 min, after which a von Willebrand factor (vWF) assay was performed on the STA-R Evolution analyzer (Diagnostica Stago, Gennevilliers, France). STA-Liatest^®^ vWF:ag kits were employed to measure vWF level. Total PAI-1 level was measured using an enzyme-linked immunosorbent assay (ELISA) with Asserachrom^®^ PAI-1 (Diagnostica Stago, Gennevilliers, France).

Heparanase procoagulant activity assay (HPA): A basic experiment of factor Xa generation was performed in the following manner, with the given concentrations being the final ones. We incubated 25 µL of the plasma, recombinant human factor VII (0.04 μM), and plasma-derived human factor X (1.4 μM) in a 50 μL assay buffer [0.06 M Tris, 0.04 M NaCl, 2 mM CaCl_2_, 0.04% *w*/*v* bovine serum albumin, pH 8.4] to a total volume of 125 μL in a 96-well sterile plate. After 15 min at 37 °C, the chromogenic substrate was added to factor Xa (1 mM). After 20 min, the reaction was stopped with 50 μL of glacial acetic acid and the level of Xa generation was determined using an ELISA plate reader (PowerWave XS; BioTek, VT, USA). Heparins were shown to abrogate the TF/heparanase complex [46]. In parallel, the same assay was performed except that fondaparinux (2.5 μg/mL) was added to the assay buffer. Bovine factor Xa diluted in the assay buffer was used to generate a standard curve. The subtraction of the first assay result from the second assay result determined Heparanase procoagulant activity [47].

Heparanase ELISA: Wells of microtiter plates were coated (18 h, 4 °C) with 2 μg/mL of anti-Heparanase monoclonal antibody 4B11 in 50 μL coating buffer (0.05 M Na_2_CO_3_, 0.05 M NaHCO_3_, pH 9.6). The plate was covered with adhesive plastic and incubated overnight at 4 °C. The next day, wells were blocked with 2% BSA in PBS for 1 h at room temperature (23 °C). Diluted samples (100 μL) were loaded in duplicates and incubated for 2 h at room temperature, followed by the addition of 100 μL polyclonal antibody 63 IgG (1 μL/mL) for an additional period of 2 h at room temperature. HRP-conjugated goat anti-rabbit IgG (1:20,000) in blocking buffer was added (1 h, room temperature) and the reaction was visualized by the addition of 100 μL chromogenic substrate (TMB; MP Biomedicals, Berlin, Germany) for 15 min. The reaction was stopped with 100 μL H_2_SO_4_ and absorbance at 450 nm was measured using the ELISA plate reader. Plates were washed four times with washing buffer (PBS, pH 7.4 containing 0.1% (*v*/*v*) Tween 20) after each step. As a reference for quantification, a standard curve was established by serial dilutions of recombinant 8 + 50 GS3 Heparanase (390 pg/mL–25 ng/mL) [48].

Reagents and antibodies: A single-chain GS3 heparanase gene construct, comprising the 8 and 50 kDa heparanase subunits (8 + 50), was purified from a conditioned medium of baculovirus-infected cells. GS3 heparanase was assayed for the presence of bacterial endotoxin (Biological Industries, Beit Haemek, Israel) using the gel-clot technique (limulus amebocyte lysate—LAL test) and found to contain <10 pg/mL of endotoxin [49].

### 4.3. Study Procedures

**Figure 3:**
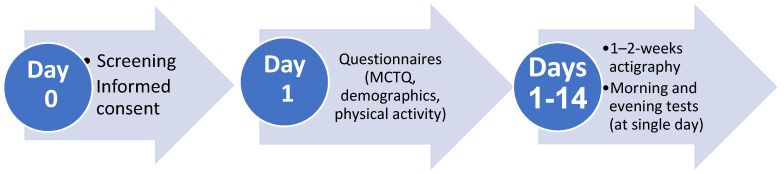
Timeline of the study.

Figure 3 presents the study timeline. Within 48 h after recruitment and informed consent, demographic and clinical questionnaires were completed and Actiwatches were handed to participants. The latter were worn on the non-dominant wrist for a 1–2-week period with concurrently completed daily sleep logs, which were used to determine time in bed. During this period of activity monitoring, participants underwent EndoPAT testing, followed by blood drawing, on a day when they were scheduled for a morning shift (day and shift workers) or an evening shift (shift workers). Morning measurements were conducted between 6:00 and 9:00 and evening measurements were conducted between 18:00 and 21:00 within 24 h, in the Thrombosis and Hemostasis Unit at Rambam Health Care Campus by a qualified nurse.

### 4.4. Statistical Analysis

Data was analyzed using SPSS software for Windows, version 28.0 (IBM Corp., Armonk, NY, USA). Means and standard deviations along with skewness and kurtosis were computed for continuous variables and frequency, and percentages were computed for categorical data. For the sleep parameters, both the means and standard deviations were averaged per participant.

Descriptive statistics were computed for demographic data, sleep parameters, and hemostatic markers of the entire sample and compared by work schedule group type using the chi-square test of independence for categorical data and *t*-test for continuous variables. Pearson’s correlation was used to assess the relationship between all sleep parameters and endothelial markers. The false detection rate (FDR) was used to correct for multiple correlations. As TST was not correlated with any of the endothelial outcomes, it was not included in the subsequent analyses.

Multiple regression was performed to predict markers of endothelial function by work schedule (day/shift), time of measurement (morning/evening), average sleep measures, separately (SEF, WASO), and two- and three-way interactions, controlling for age. Models were performed for morning and evening endothelial function measurements separately, unless no significant difference was found between the two time points. Statistical significance was set at *p* < 0.05.

## 5. Conclusions

Our findings underscore the importance of adequate sleep in maintaining vascular health. The observed associations between sleep measures and endothelial function and coagulation activation suggest a possible pathway through which sleep disruption may contribute to further cardiovascular risk. Further research is needed to elucidate these mechanisms and explore potential interventions to mitigate vascular health risks associated with poor sleep.

## Figures and Tables

**Table 1:** Sleep parameters by type of work schedule.

Sleep Parameters (Mean Participant)	All (N = 100)	Shift (N = 51) (N = 51)	Day (N = 49) (N = 51)	t	*p* *
	Mean ± SD	Mean ± SD	Mean ± SD		
TST (hours)	6.48 ± 0.82 (4.44–9.40)	6.42 ± 0.93 (4.44–9.40)	6.53 ± 0.70 (5.00–8.41)	−0.64	0.411
SEF (%)	85.7 ± 3.8 (78.6–95.4)	84.7 ± 4.0 (78.6–93.4)	86.8 ± 3.2 (79.8–95.4)	−2.85	0.005
WASO (minutes)	33.5 ± 10.3 (11.6–55.9)	35.4 ± 11.4 (11.6–55.9)	32.5 ± 8.6 (11.9–53.6)	1.89	0.06

TST—total sleep time. SEF—sleep efficiency. WASO—wake after sleep onset. * uncorrected *p* values.

**Table 2:** Correlations between sleep parameters and endothelial markers for whole sample.

		LnRHI		PAI-1		VWF		Heparanase ELISA		HPA	
*Time of Measurement*		Morn	Even	Morn	Even	Morn	Even	Morn	Even	Morn	Even
TST	Pearson, r	0.028	0.046	−0.096	−0.005	−0.073	−0.086	−0.107	−0.106	−0.044	−0.158
	p	0.779	0.647	0.343	0.960	0.471	0.397	0.288	0.292	0.661	0.116
SEF	Pearson, r	0.347	0.419	−0.691	−0.517	−0.090	−0.160	−0.446	−0.406	−0.453	−0.425
	p	<0.001	<0.001	<0.001	<0.001	0.373	0.113	<0.001	<0.001	<0.001	<0.001
	FDR p	0.0013	0.0012	<0.001	<0.001	0.373	0.125	0.0012	0.0013	0.0012	0.0013
WASO	Pearson, r	−0.325	−0.226	0.559	0.329	0.028	0.070	0.300	0.321	0.179	0.198
	p	0.001	0.024	<0.001	0.001	0.784	0.487	0.002	0.001	0.075	0.049
	FDR p	0.0025	0.04	0.0025	0.0025	0.784	0.541	0.004	0.0025	0.094	0.07

TST—total sleep time. SEF—sleep efficiency. WASO—wake after sleep onset.

## Data Availability

All data generated or analyzed during this study are included in this published article.
